# ENISA: 5G design and architecture of global mobile networks; threats, risks, vulnerabilities; cybersecurity considerations

**DOI:** 10.12688/openreseurope.15219.2

**Published:** 2023-03-29

**Authors:** R. Andrew Paskauskas

**Affiliations:** 1 Cybersecurity Group, Lithuanian Cybercrime Center of Excellence for Research, Training and Education (L3CE), Vilnius, 08303, Lithuania

**Keywords:** ENISA, cybersecurity, 5G design, 5G architecture, threats, vulnerabilities, risks, mobile networks

## Abstract

*Abstract *—The literature on 5G design and architecture numbers in the hundreds of thousands, which makes analyzing this vast corpus of technical knowledge impossible within the scope of a single article. A rigorous literature scan has revealed investigations of various specific 5G components, or specific aspects of 5G design, architecture, or security, but none that are comprehensive in scope, encompassing all of the aforementioned categories, or that take into account the associated vulnerabilities, threats and risks to the basic 5G infrastructure.

In this sense the 5G framework advocated by The European Union Agency for Cybersecurity (ENISA) in its comprehensive report is singular in relation to the extensive literature associated with the 5G domain and the fragmented character of scientific reporting related to 5G technology.

It is the purpose of this article to go beyond the existing literature and examine in depth the details of the ENISA 5G Threat Landscape Report and reveal ENISA’s painstaking efforts to stand out among other leading-edge players in the 5G arena and achieve its strategic aims of integrating cybersecurity considerations with threats, risks, and vulnerabilities into an architecture of 5G right from the start of the design and development process.

In formulating such a framework, ENISA has set the stage for standardization of cybersecurity considerations in relation to 5G design and architecture that may be considered a first approximation towards best practice in the field.

ENISA’s role in the European Union as a leader in setting the pace of development of 5G networks is acknowledged in EU’s legislation and its directives. Significantly, its strategic direction targets future implementations of 5G networks by vendors, operators, and practitioners. This should equip EU with the necessary resilience to withstand hybrid threat onslaughts on its Pan-European network, a topic to be dealt with in full in a follow-on paper.

## Plain language summary

The European Union Agency for Cybersecurity (ENISA) stands out among other leading-edge players in the 5G arena because it has made a strategic decision to integrate cybersecurity considerations with threats, risks, and vulnerabilities into an architecture of 5G right from the start of the design and development process. ENISA’s ‘THREAT LANDSCAPE FOR 5G NETWORKS’ (Dec 2020) offers a strategic framework for the design and architecture of 5G generic frameworks. 5G is a fifth-generation mobile phone communications standard. It is a successor to 4G and has promised to be faster than previous generations while opening up new uses for mobile data.

## Introduction

The strategic direction of The European Union Agency for Cybersecurity (ENISA) in relation to 5G is outlined in detail in a lengthy document entitled 'ENISA THREAT LANDSCAPE FOR 5G NETWORKS’ December 2020 ^1^. This publication is part of a series of annual reports on the state of the cybersecurity threat landscape, identifying prime threats, major trends observed with respect to threats, threat actors and attack techniques, including relevant mitigation measures. As of October 2022, 10 editions have appeared.

The Threat Landscape report under consideration here assembles existing threats and vulnerabilities found in a wide range of open-source resources and research papers that cover state-of-the art 5G technologies and address security requirements related to 5G network functions. Taking all of this into account, ENISA arrives at practicable and workable conclusions, at the same time highlighting technical and operational weaknesses or omissions in its approach.

As will be seen, however, we can compensate for these drawbacks by deferring to the work of other associations or organizations, and to the scientific and technical literature in the field. This literature however is too vast to deal with adequately in the present paper, but we can point to recent studies containing essays of over 300 pages ^2^, or close to 1,000 pages ^3^, that encompass the wide terrain of cybersecurity, wireless networks, cloud computing, IoT, vulnerabilities, threats, risk mitigation, standards or various forms of forensics applied to these domains.

The detailed technical components of 5G networks emerging from the collected material and integrated into the ENISA report have been highly influenced by the standards, good practices and specifications contained in the 3rd Generation Partnership Project (3GPP).

ENISA has incorporated the security features of the 3GPP specification contained in Release 16 ^4^ into its 5G design and architecture, and has integrated vulnerabilities, threats and risks, alongside cybersecurity considerations, into a unified framework that anticipates forthcoming 3GPP Releases ^5^ and that will pave the way for viable future implementations of this emerging technology on a wide scale by vendors, operators, and a host of players in the 5G domain.

3GPP does not work alone. It takes into account the work of various telecommunications standards organizations ( Table I) ^i^ and provides their members with foundational outputs that define 3GPP technologies.

**Table I.** Global standards organizations working with 3GPP. Organisational Partners representing Asia, Europe and North America are charged with overseeing the work of Technical Specification Groups and transposing reports and 3GPP specifications into formal deliverables (i.e., standards).

*Organization*	*Acronym*
The Association of Radio Industries and Businesses, Japan	ARIB
The Alliance for Telecommunications Industry Solutions, USA	ATIS
China Communications Standards Association	CCSA
The European Telecommunications Standards Institute	ETSI
Telecommunications Standards Development Society, India	TSDSI
Telecommunications Technology Association, Korea	TTA
Telecommunication Technology Committee, Japan	TTC

As well, numerous market representation partners provide advice, offering a consensus view of market requirements such as services, features and functionality to 3GPP working groups. A list of twenty-five such partners ^ii^ includes Global System for Mobile Communications Association (GSMA), ^iii^ an industry organization that represents the broader mobile community, as well as the interests of mobile network operators worldwide, and involves its members in industry programs, working groups and industry advocacy initiatives to drive the future of mobile technology services, solutions and specifications. ^iv^ For example, GSMA together with 3GPP have forged the Network Security Assurance Schemes (NESAS) standards, and outcomes from working groups have resulted in equipment vendors such as Ericsson, Huawei, Nokia and ZTE demonstrating full compliance with this industry-wide security assurance framework. ^v^

As described by ENISA, GSMA has also been tasked with establishing standards for eSIMs ^vi^ to ensure that security, reliability and interoperability functionalities are preserved. Regarding the use of eSIMs in 5G, for example, GSMA will advise on issues where network security policies aimed at preventing attacks need to be implemented at a technical level [ 6, p. 33]. 

## ENISA: Design and architecture

The ENISA Threat Landscape consists of design and architecture elements mapped to vulnerability groups in the form of 'Zoom-ins', as well as to a more detailed elaboration in the Annexes of vulnerabilities and risks threatening the security of the overall structure of the 5G framework.

### 5G network design and architecture

This section of the ENISA Threat Landscape consists of thirteen segments ( Table II), each of which delves into the details of the design and architecture of 5G technologies, primarily based, but not exclusively, on Release 16 of the 3GPP specification. ^vii^

**Table II.** 5G network design and architecture.

*Principal components*
5G use cases
Generic 5G architecture
Core network architecture
Network slicing (NS)
Management and network orchestrator (MANO)
Radio access network (RAN)
Network function virtualization (NFV) – MANO
Software defined network (SDN)
Multi-access edge computing (MEC)
Security architecture (SA)
5G physical infrastructure
Implementation options / migration paths
Process MAP

The first two segments are of general import. These are:

i) 5G use cases, and ii) generic 5G architecture. The following seven design and architectural elements are described in the form of “Zoom-ins”, which provide general summaries of each element, along with associated technical enhancements and an assessment of noteworthy security considerations. The remaining four categories (bottom of Table II) depart somewhat from the previous set of seven but pay similar attention to the importance of integrating security features with the detailed design and architecture of the 5G framework.

The more exhaustive features of ENISA’s 5G design and architecture reveal where the framework draws from 3GPP’s Release 16 specification.

For example, the “5G Use Cases” segment includes the principal mechanisms of 5G functionality: enhanced mobile broadband (eMBB) which handles services having steep requirements for bandwidth; ultra-reliable low latency communication (uRLLC) for assisted or automated driving; and machine type communications (MTC) for smart cities; along with the addition of new use-cases and advances in technical specifications.

In segment “Generic 5G Architecture”, the Management and Orchestration (MANO) component is made up of two parts: i) 3GPP MANO—network slicing management, and ii) ENISA’s addition to the architecture, its non-3GPP MANO (i.e., network function virtualization-MANO, software defined network-controller, operation support system).

An important item in Release 16 includes enhancements in the 5G core network architecture, especially in the support for *enhanced* ultra-high reliability low latency communications (e-uRLLC), in which the marked influence of the 3GPP formulation clearly stands out ^7^. ENISA explains that its *ad hoc* expert groups include skilled individuals, experts in 5G. In turn, each individual chosen by 3GPP from its long list of stakeholders and partners takes on a role as an interface between ENISA and 3GPP. In the case of “Enhancement of URLLC support in the 5G Core network” (Section 5 in 7) we note the list of ten work items, each of which is associated with a *Rapporteur* from a 3GPP industry partner – the majority of *rapporteurs* in this instance being from Huawei. Detailed summaries for the e-URLLC spec are contained in 7.”

It is important to understand that the e-URLLC specification is just one of close to 115 specs, all of which are supported by 100s upon 100s of technical documents.

## ENISA: 5G vulnerabilities

For each of the thematic elements of the design and architecture discussed previously, vulnerability groups are described briefly and accompanied by highlights of the assessed weaknesses. Of crucial significance here is the fact that the vulnerabilities identified will be mapped to the security considerations formulated for each of the “Zoom- ins” discussed in the “5G network design and architecture section”. Also included are identified cyberthreats that may lead to exploitation of vulnerabilities, along with pointers to the relevant security protection measures contained in the ENISA 5G Toolboxes, as well as references to the relevant literature. Of interest too, is the fact that vulnerability groups presented here target technical experts who may be interested in gaining an overview of weaknesses of various technical components of the ENISA 5G framework.

## ENISA annexes A – M

Here, a “5G Asset Mind Map” is introduced in Annex A ( 1, p. 123) which is a powerful visual representation of the assets that map to the 5G elements of the framework. A high-level view is revealed in the ENISA document, but readers should consult the user-friendly visualization. ^viii^ Highlights of specific assets contained in the mind map and their relationship to 5G Security Architecture will be discussed later on.

Annex B deals with the intersection between the threat taxonomy of the International Telecommunication Union (ITU) and that of ENISA. With particular attention to the “Nefarious activity/abuse” category, as a starting point, a detailed itemization of a large set of threats linking the two taxonomies is put forward. This version of ENISA's Threat Taxonomy is depicted in another highly informative and readable visual representation ( 1, p. 128).

Each Zoom-in of Section 3, as well as the Vulnerability Groups of Section 4, are dealt with further in corresponding Annexes (C-M), which fully describe the vulnerabilities associated with the complete set of 5G Design and Architecture elements. These details are provided in the form of an extensive set of tables throughout C-M. Lists and detailed descriptions of vulnerabilities are included, along with relevant assets, threats exploiting the vulnerability, security controls to remove/reduce the exploitation in question, and stakeholder responsible for the implementation of controls, as well as references to relevant sources.

Associated risks are dispersed periodically throughout this section and primarily identified in the table columns entitled “Description” and “Security Requirements”.

## Key standards organizations

National/international standards organizations are hard pressed to compete with the ENISA/3GPP formulation of 5G. With the exception of several peripheral and isolated contributions to the 5G specification, these standards entities continue to be engrossed in dealing with sets of discrete standards elements of legacy systems. The important standards organizations are listed in Table III.

**Table III.** Principal standards organizations world-wide.

*International*	*Acronym*
International Organization for Standardization ISO - International Organization for Standardization	ISO
International Electrotechnical Commission Homepage | IEC	IEC
International Telecommunication Union ITU: Committed to connecting the world	ITU
ITU: Telecommunication standardization ITU Telecommunication Standardization Sector	ITU-T
ITU-R: Radiocommunication standardization Welcome to ITU-R	ITU-R
*Global System for Mobile Communications Association* GSMA | Brief History of GSM & the GSMA - About Us	GSMA
*European*	*Acronym*
European Committee for Standardization Home | CEN	CEN
European Committee for Electrotechnical Standardization About CENELEC - CEN-CENELEC (cencenelec.eu)	CENELEC
European Telecommunications Standards Institute ETSI - Welcome to the World of Standards!	ETSI
*United States of America*	*Acronym*
National Institute of Standards and Technology National Institute of Standards and Technology (nist.gov)	NIST
American National Standards Institute American National Standards Institute - ANSI Home	ANSI
National Telecommunications & Information Administration National Telecommunications and Information Administration | United States Department of Commerce (doc.gov)	NTIA
InterNational Committee for Information Technology Standards Home - INCITS	INCITS
IEEE Standards Association IEEE SA - The IEEE Standards Association - Home	IEEE SA

The three European Standards Organizations, CEN, CENELEC and ETSI have been established by a transparent, open and consensual process involving all interested stakeholders. These standards are key to the proper functioning of the Single European Market.

More specifically, the relationships among members of ISO, IEC, CEN, and CENELEC ensure that the interests of European businesses and other stakeholders are recognized at the international level ( Table III). For example, the ISO-CEN/Vienna Agreement provides a means for ISO standards to become CEN standards and vice versa. The same holds for the IEC- CENELEC (CLC)/ Frankfurt Agreement. ^ix^

But these organizations are lacking in approaches that would embrace holistic strategies that are all-encompassing in relation to emerging technologies. For example, although CEN (European Committee for Standardization) and CENELEC (European Committee for Electrotechnical Standardization) have established the CEN-CENELEC Joint Technical Committee 21 ‘Artificial Intelligence’, based on the recommendations presented in the CEN-CENELEC response to the EC White Paper on artificial intelligence (AI), ^x^ and the German Standardization Roadmap on AI ^8^, there is no comprehensive analysis of 5G or IoT to be found in CEN-CENELEC major initiatives.

The German approach in particular continues to echo the preferred strategy of international organizations. Its roadmap simply lists a wide range of discrete standards elements as representative of its AI framework with no regard to the design or architecture of the entire complex. All told there are over 700 references to ISO/IEC/IEEE/ETSI/IETF/ITU- T/CEN/CENELEC, either separately or in combination. Furthermore, in Table 10: Existing standards and specifications on AI, the label “Brief description with possible relevance to AI” ( 8, p. 144) is employed which evokes a lack of clarity in the role a standard may assume in the overall AI scheme. Similarly, “architecture” is employed without regard to an all-encompassing framework.

However, we cannot exclude entirely the role played by at least several of the international organizations in ENISA’s formulation of its 5G framework. The ambitious nature of this initiative can be gleaned from its major accomplishment in putting forward a comprehensive design and architecture of 5G, based on the details of the 3GPP specification. But that accomplishment has also been achieved by taking into account more than a handful of the standards organizations listed in Table III above, as the following statement to be found in ENISA Threat Landscape clearly reveals ( 1, p. 11)

A detailed technical and operational vulnerability analysis has been performed for the components of the 5G architecture. This analysis takes into account the threats exploiting those vulnerabilities and the controls reducing exposure to these threats, as defined by international organizations (3GPP, ETSI, GSMA, ISO, ITU, NIST).

### Discrete aspects of international standards

The European Commission's study on the taxonomy of cybersecurity research domains refers to ISO/IEC standards throughout its report, at least 135 times, yet provides the caveat that these standards describe very specific aspects of the cybersecurity domain, not the cybersecurity ecosystem as a whole. Moreover, vulnerabilities, risks and threats are referred to as discrete elements within working groups or specific cyber components and not within a global design and architecture framework ^9^.

To grasp more fully the issue of specificity with respect to the ISO/IEC approach to standards one need only consider a few examples that address vulnerabilities. These are:

- ISO/IEC 29147:2018 Information technology - Security techniques - Vulnerability disclosure
- ISO/IEC 30111:2019 Information technology – Security - Vulnerability handling processes
- ISO/IEC 24772:2019 Programming languages – avoiding vulnerabilities in programming languages

These examples illustrate particularly the difficulty of undertaking serious research in the international standards domain – surprisingly, each complete standard will run up approximately USD 175, and there are 100s of discrete standards in question to reckon with.

Significantly, some international organizations have broken away from relying on discrete standards. One example is the ongoing work of the International Telecommunication Union (ITU) which through its ITU Radiocommunication (ITU-R) Sector has defined a vision and roadmap for network devices and services, including 5G mobile development, as for example, its ‘Recommendation ITU-R M.2083-0 (09/2015) IMT Vision – Framework and overall objectives of the future development of IMT for 2020 and beyond.’ ^xi^: Here, ITU-R has defined the 5G vision and high-level requirements for 5G. ^xii^ In other words, 3GPP uses the ITU-R recommendations as a basis for defining the radio access technologies used in cellular networks such as 5G. Thus, ITU-R has been highly influential in the efforts exhibited by the ENISA/3GPP collaboration. 

There are numerous players in the vulnerability capture domain whose work is exemplified in the following ways: The National Cyber Security Centre (NCSC), Netherlands, has put out a general guideline for responsible vulnerability disclosure; ^xiii^ The Common Vulnerability Scoring System (CVSS) ^xiv^ defines a standardized approach for describing and scoring vulnerabilities, according to risk and severity levels; The Common Vulnerabilities and Exposures (CVE) ^xv^ list provides an internationally recognized standard for naming and cataloguing known cybersecurity vulnerabilities, along with publicly released advisories; the Software Vulnerability Disclosure in Europe report ^xvi^ was put out in 2018 by the Centre for European Policy Studies (CEPS).

This work has merit but unless integrated into an overall design and architectural framework that can offer the resilience necessary to mitigate risks, organizations will be at a loss as to how to counter threat onslaughts, especially if these arise from a wide variety of hybrid sources ^10^. The robust nature of such frameworks called for in this instance is exactly what 3GPP and partners can offer.

### Vulnerabilities: GSMA, 3GPP and ENISA

As ENISA points out, GSMA’s Vulnerability Disclosure Program provides a framework that sets clear expectations for constructive engagement by those concerned with providing input for standardization work, meeting security requirements, and mitigating risks associated with known vulnerabilities ( 1, p. 69).

In conjunction with other Standards Development Organizations and related bodies, 3GPP has put in place a mechanism by which individuals or organizations can make use of the GSMA Coordinated Vulnerability Disclosure (CVD) procedure ^xvii^ by using an online submission form. ^xviii^ Thus, suspected or proven vulnerabilities caused by errors, omissions or ambiguities in the technical specifications can be captured, particularly those which could give rise to security breaches or loopholes that might compromise 3GPP network components, terminal equipment connected to those networks, or to other interworking mechanisms or equipment. The extent to which this procedure is able to accommodate the sum total requirements of the emerging 5G complex, especially in relation to ENISA’s framework, is promising.

**Figure 1.**
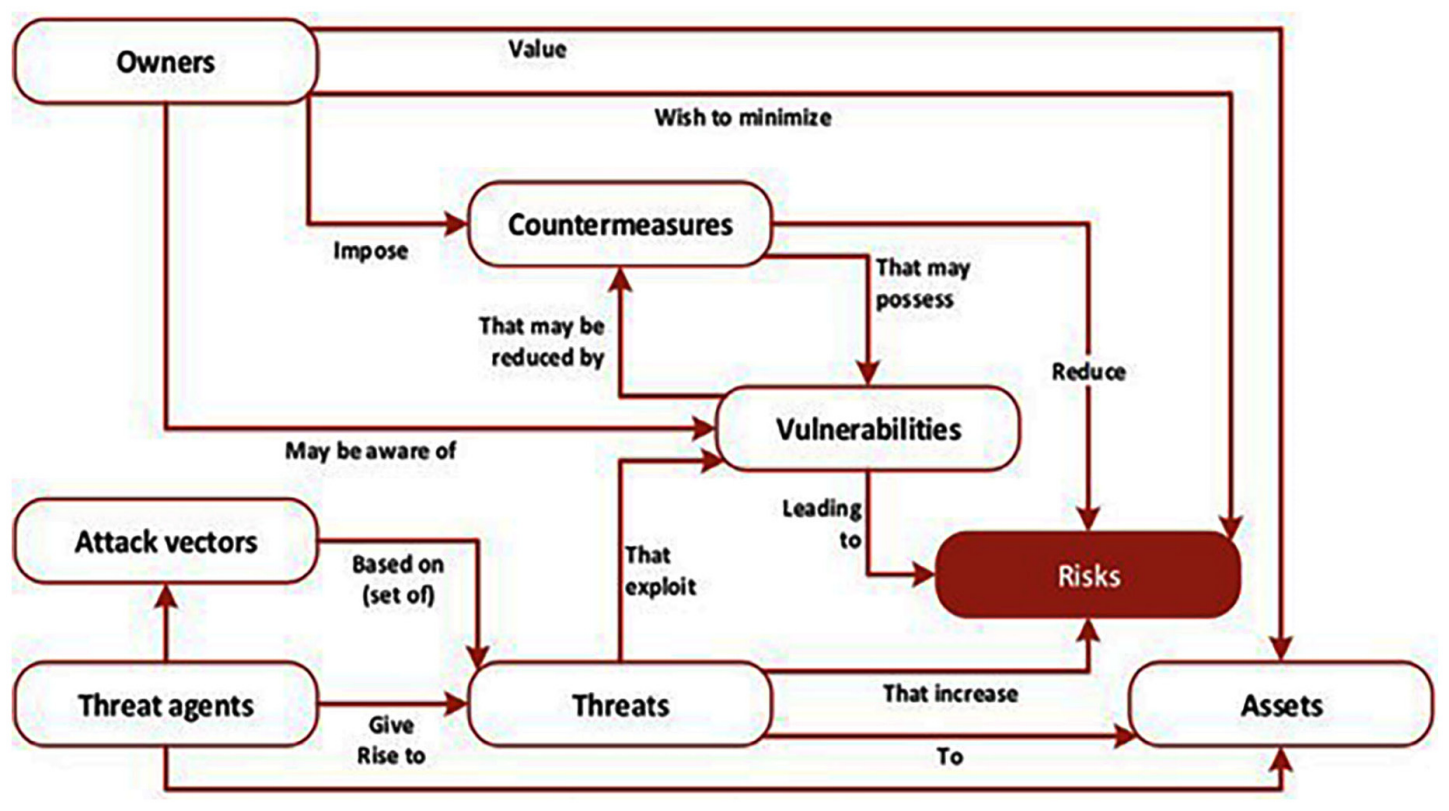
The multifaceted aspects of risks. The various elements of cyberthreats, vulnerabilities, assets and their relationship to risks are depicted in the figure; all are woven into the design and architecture of the ENISA framework. © European Union Agency for Cybersecurity (ENISA), 2020.

Significantly, this is where the power of ENISA’s recommendations comes into play and its further debt to an international standards organization is acknowledged. The methodology as visualized by ENISA and depicted in Figure 1. ( 1, p. 12) is based on the ISO 27005 standard and connects vulnerabilities directly with threats and risks in relation to owners and assets, attack vectors, threat agents, and countermeasures, and these are integrated into the overall design and architecture of the ENISA framework.

What is more, the ENISA analysis goes deeper into key structural aspects of its framework ( Table IV) by providing an elaborate assessment of the role of asset classification, the CIA triad (confidentiality, integrity, availability), as well as a taxonomy of threats and threat agents ( 1, pp. 93–119).

**Table IV.** Classification of assets and threats.

*Section 5. Assets*
Asset classification and mapping
New asset categories
Asset Classification and the CIA triad
Relevance of assets throughout lifecycle
*Section 6. 5G Threats*
Taxonomy of threats
Threat map
*Section 7. Threat Agents*

ENISA together with the European Commission also offer a portfolio of tools and related documents associated with risk mitigating measures that practitioners can apply to specific vulnerabilities in order to enhance resilience against unwanted threats.

## Hyperconnectivity, IoT, industrial IoT

The present-day pervasive interest in Internet of Things (IoT) is dealt with sparingly in the ENISA report. As a result, the European Commission’s preoccupation with the emerging concept of hyperconnectivity may serve as a viable complement to ENISA’s treatment of 5G in relation to IoT, cellular IoT, or industrial IoT.

### 5G, hyperconnectivity, IoT

Hyperconnectivity embraces a multitude of uses which tend to be unruly, including multiple devices supporting various means of communication such as person-to-person, person-to-machine or machine-to-machine that remain constantly connected to social networks and wide-ranging streams of information. These may include email, instant messaging, phone-based communication, face-to-face contact or Web 2.0 information services. ^xix^

The European Commission (EC) has outlined the emerging trends in hyperconnectivity, including the role of computing resources virtualization and network slicing. Internet of Things (IoT) especially is considered a strategic enabler of a hyperconnected society, representing an infrastructure of networks and devices that will soon track in the billions.

Current research suggests that viable implementations of IoT imply that effective 5G capacities to manage virtualization and network slicing are in play ^11^, which we found to be the case earlier in ENISA's “Generic 5G Architecture” ( Table II and subsequent discussion).

EC claims that IoT suffers from a lack of interoperability across platforms resulting in data silos that are costly and markets with limited potential. Indeed, a similar situation existed when there were competing non-interoperable networking technologies prior to the birth of the Internet itself. The World Wide Web Consortium (W3C) seeks to counter the fragmentation of IoT implementations by expanding the use of existing standardized Web technologies into a Web of Things (WoT). ^xx^

Hyperconnectivity and IoT in all its forms are set to explode globally, driven by 5G communication infrastructure and higher-volume and lower cost devices. ^xxi^ As GSMA reports, by 2025, there could be up to 1.4 billion connections worldwide, a significant increase from about 200 million 5G connections in 2021. ^xxii^ Hence, the crucial importance of ENISA’s interest in supporting and engaging in current and future initiatives in this domain.

### Enhanced ultra-reliable low-latency communication

In the design and architecture section above, we noted that our discussion of the ENISA/3GPP Release 16 undertaking placed great weight on increasing support for enhanced ultra-reliable low-latency communication (e-URLLC).

To elaborate more fully, the aim of this effort on ENISA’s part is to strengthen the 5G Core network mechanisms, especially to reduce latency, to guarantee session continuity and to increase reliability. In addition, physical layer enhancements have been introduced into the 5G New Radio (NR) component, as were enhancements and support of NR Industrial IoT mechanisms such as improved handling of Time Sensitive Networking (TSN) capabilities for accurate synchronization of time dependent factory processes as would be required for industrial applications. All of this is consistent with current research in the field ^12^.

### 5G and industrial IoT 

As a further complement to the ENISA framework, the 5G Alliance for Connected Industries and Automation (5G ACIA), a product of the German Electrical and Electronic Manufacturers’ Association (ZVEI), may also prove to be a viable addition to the ENISA initiative with reference to the IoT domain, especially Industrial IoT.

Germany's impressive work in “Designing 5G for Industrial Use” has been highly influenced by the 3GPP specification, including GSMA. ^xxiii^ Indeed, 5G ACIA is intent on developing 5G capabilities that will pave the way for perfecting Industry 4.0 communication and Industrial IoT connectivity. Its principal area of focus deals with the overall architecture of future 5G-enabled industrial infrastructures, including integration concepts and migration paths towards sophisticated industrial applications. It also works with standardization bodies to evaluate relevant 5G technologies, but clearly the 3GPP consortium is its principal point of influence when considering its vast array of concerns related to the 3GPP specification as applied to the industrial domain.

## Cybersecurity considerations

In this concluding segment on cybersecurity, we will continue highlighting the work of 3GPP and add to this the efforts of the principal technical work group on the architecture of security called Service and Systems Aspects 3 (SA3). To be sure, SA3’s influence on ENISA’s comprehensive strategic approach to cybersecurity will prove to be of paramount importance.

As discussed earlier, the national and international standards organizations approach their respective tasks in a discontinuous fashion tackling specific standards within a very narrow context, and thus fall prey to falling behind the important work undertaken by global initiatives such as 3GPP/SA3 and its industry partners that have so dramatically influenced the efforts of ENISA. Consider just a few examples out of many where this argument applies.

### Europe

Europe’s “5G Action Plan” (2016) ^xxiv^ and the “European Electronic Communications Code (EECC)” (2018) ^xxv^ together represent an attempt to foster the competitiveness of European industry. In fact, ENISA has published its own version of the EECC ^13^, as well as a supplement devoted to outlining how the code applies in the context of 5G ^14^. An important offshoot of the action plan has been the establishment of the 5G Infrastructure Public Private Partnership (5G PPP). ^xxvi^ With its impressive array of industry and association partners, and numerous European cities tasked with various testing procedures, 5G PPP has embarked upon a wide range of technical projects and acknowledges the important role that 3GPP has played in providing a general framework for a 5G architecture. Its concern with verticals is an area where it complements the ENISA framework as it is clearly intent on contributing to the task of implementing and creating new markets related to smart cities, e-health, intelligent transport, education, entertainment, and media.

5G PPP has also joined forces with the 5G Infrastructure Association (5G IA). A position paper advocating a European Partnership under Horizon Europe called “Smart Networks and Services” (SNS June 2020) ^xxvii^ addresses the challenges of cybersecurity to a certain degree and points to numerous 5G features that need to be considered, but design and architectural concerns are dealt with at a high level and lack an all-encompassing strategic approach to do with the key issues of technical implementation. Vulnerabilities are also not identified in marked contrast to the ENISA approach. However, 5G IA, has had a pivotal role in transforming 5G initiatives into an SNS framework, and plans to entice European researchers and practitioners to develop and implement ‘6G’ technologies to support future digital services over the next decade. ^xxviii^ This latter effort is of consequence as results of work on 6G tend to reveal serious weaknesses of 5G ^15^.

### The international arena

If we turn our attention to the international stage, we can point to 3GPP’s principal working group on security, SA3, which is tasked with defining requirements and specifying the architecture and protocols for security and privacy in 3GPP systems. SA3 is especially concerned with 3GPP enhancements to IoT and vertical industries. As before, the work is undertaken in collaboration with a wide range of industry partners.

As far as standards for mobile networks are concerned, ENISA’s treatment of the challenge is sporadic in the Threat Landscape report. However, the Agency takes on a more rigorous approach in its report on 5G cybersecurity standards ^16^. In its analysis of the complexities involved – mitigation of technical risks, for example – ENISA undertakes an assessment of the coverage of the standards, specifications and implementation guidelines with respect to 5G, as well as identification of gaps in standardization. A principal output of the report gives rise to the formulation of recommendations on standardization in the area of 5G security.

Still, as Ericsson, one of the leaders in the field, has argued, there is no one single security standard. Ericsson succinctly summarizes the argument this way: ^xxix^

The main standardization organization for mobile networks is 3GPP, and the security for 3G through 5G has been defined in the security group SA3. The security architecture, as defined by 3GPP SA3, in turn comprises security solutions from several different standardization organizations. The IETF defines security protocols such as IPsec, EAP, and TLS which are incorporated in the 5G security architecture. A 5G network is built using cloud and virtualization technologies, and ETSI ISG NFV defines security for network functions virtualization (NFV). Crypto solutions such as AES are standardized by NIST, and the recently approved NESAS framework for security assurance is a joint effort between 3GPP SA3 and GSMA (See Table V).

**Table V.** 5G security related terminology.

*Acronym*	*Group/protocol/organization* */function/standard*
Internet Engineering Task Force	IETF
Internet Protocol Security	IPsec
Extensible Authentication Protocol	EAP
Transport Layer Security	TLS
European Telecommunications Standards Institute	ETSI
Industry Specification Group	ISG
Network Functions Virtualization	NFV
National Institute of Standards and Technology	NIST
Advanced Encryption Standard	AES
Network Equipment Security Assurance Scheme	NESAS

In addition, consider that 3GPP/SA3 relies heavily on industry for defining its security features, just as it had for its design and architecture specifications discussed previously. Here, Huawei, Nokia and Ericsson standout among others. In turn, we note too that these companies rely on the 3GPP/SA3 specification to define their own architectures.

It is also noteworthy that Ericsson has alluded to the fact that the 5G network relies on cloud and virtualization technologies, and that ETSI ISG NFV defines security for network functions virtualization (NFV), which is identical to the way the ENISA formulation has treated both virtualization and possibilities of adapting NFV to the cloud and employing software and virtualization techniques to create novel architectures ( 1, pp. 36–37). In fact, ENISA has gone even further with an elaborate study of 166 pages on NFV as applied to 5G. Its objectives are worth quoting in full:

This ENISA study aims at underlining and analysing the security challenges related to 5G NFV. The main objectives are to identify challenges and best practices to ensure the security of 5G NFV, while mapping the relevant security challenges, vulnerabilities, attacks scenarios, assets and best practices [ 17, p. 7].

When all aspects and components are put together, ENISA’s attempt to form the security standard for 5G can be considered as a first approximation towards best practice in the field. Again, we emphasize, the security features of the ENISA formulation are embedded in the design and architecture of the 5G framework.

To illustrate the power of ENISA’s approach we turn to the discussions in its Threat Landscape document, Sections 3 and 5.

### Security architecture / asset classification-mapping

**Figure 2.**
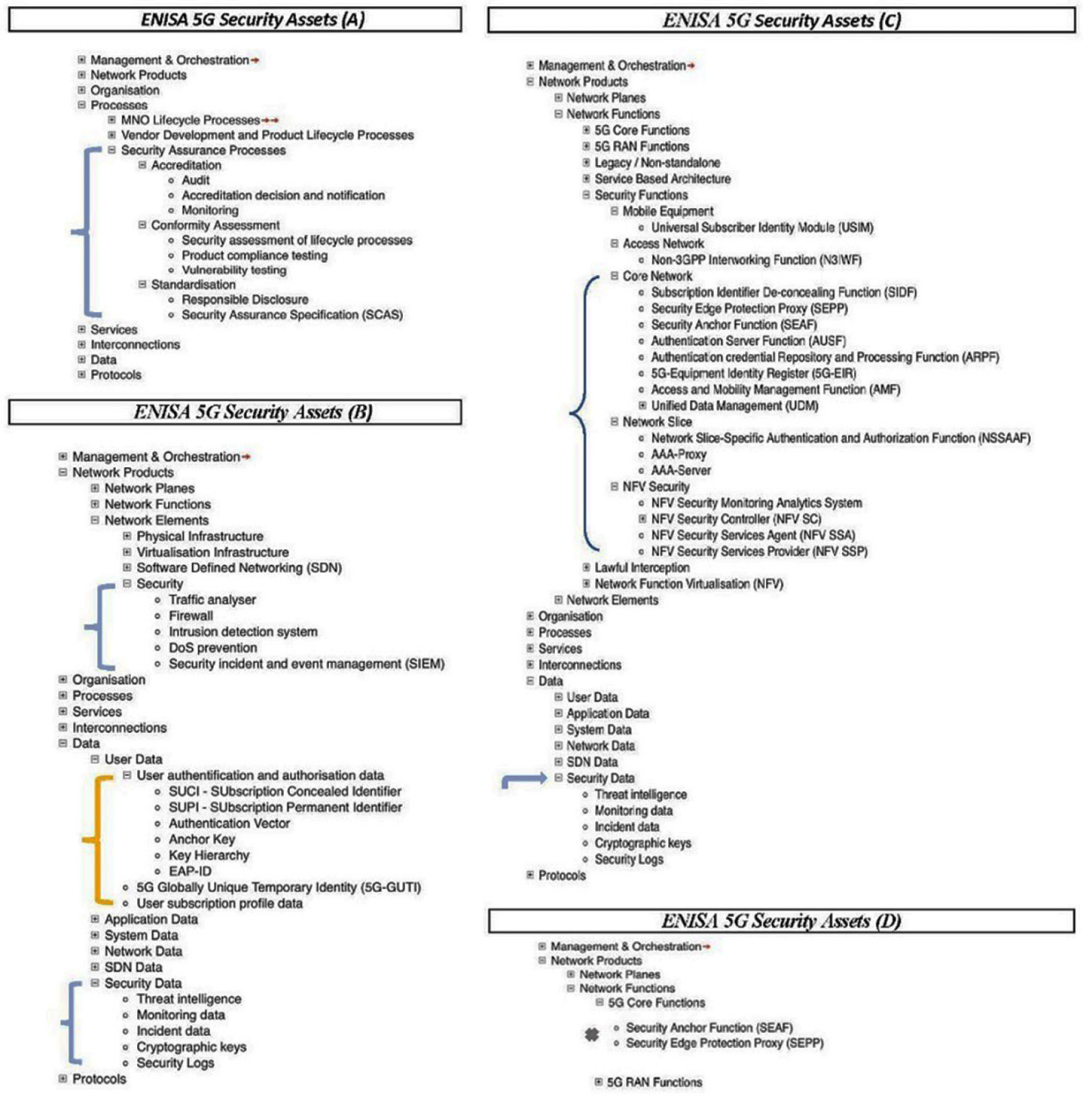
Excerpts from ENISA annex A, as visualized by ENERSEC, focusing on 5G security features. Network Functions under major asset Network Products (C) contains category Security Functions which consists of five sub-categories containing seventeen additional security elements, which are shown above, where they are delineated as follows: i) Mobile Equipment: Universal Subscriber Identity Module (USIM); ii) Access Network: Non-3GPP Interworking Function (N3IWF); iii) Core Network: SIDP, SEPP, SEAF, AUSF, ARPF, 5G-EIR, AMF, UDM,; iv) Network Slice: Network Slice-Specific Authentication and Authorization (NSSAAF), AAA-Proxy, AAA-Server,; and v) NFV Security whose elements NFV Security Monitoring Analytics System, NFV SC, NFV SSA, and NFV SSP make up a good part of the components depicted in the Figure 4 (bottom), as do many of the assets identified above in ‘iii)’ representing integral components of ENISA’s 5G Security Architecture as depicted in Figure 4 (top). Moving down to major asset Data (B), the security asset elements associated with user authentication and authorization (SUCI, SUPPI, Authentication Vector, Anchor Key, Key Hierarchy, EAP-ID) are all integrated as well into ENISA’s 5G Security Architecture depicted in Figure 4 (top). The mind map is contained in the ENISA Threat Landscape Report ( 1, p. 123). © European Union Agency for Cybersecurity (ENISA), 2020; and browsable Mind Map creators: ENERSEC, Bucharest, Romania ( https://www.enersec.net/).

**Figure 3.**
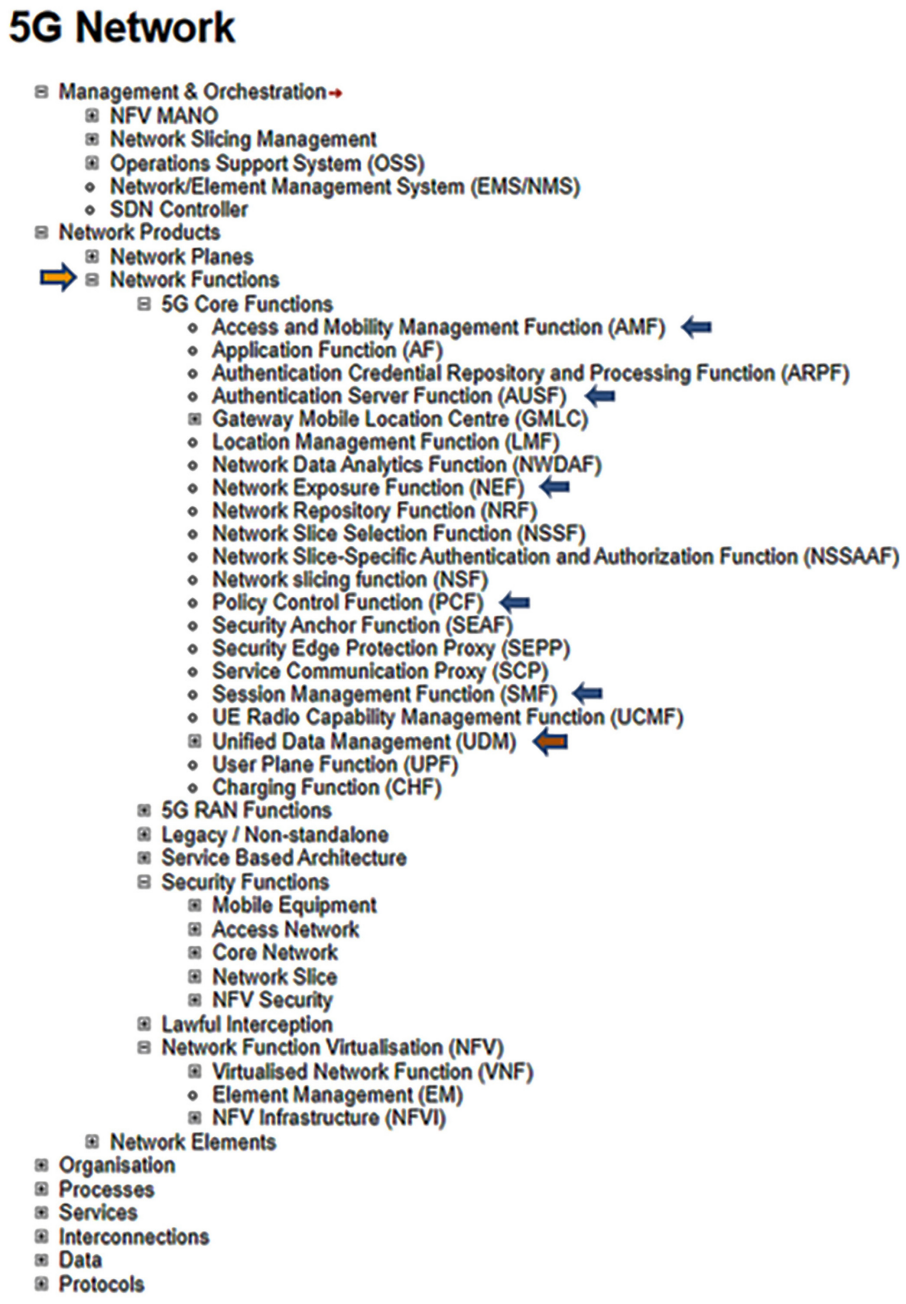
Excerpts from ENISA Annex A, focusing on network functions. ENISA Threat Landscape Report ( 1, p. 123). The assets which are marked with arrows (AMF, AUSF, NEF, PCF, SMF, UDM) represent the most prominent elements demarcated in the Unified Data Repository, UDR Data Storage Architecture. © European Union Agency for Cybersecurity (ENISA), 2020; and browsable Mind Map creators: ENERSEC, Bucharest, Romania ( https://www.enersec.net/).

The painstaking integration of cybersecurity elements into the design and architecture of the ENISA framework can be appreciated by carefully examining the wide range of 5G assets depicted in Annex A, 5G Asset Mind Map, of the Threat Landscape document and broken down in Figure 2 and Figure 3 ( 1, p. 123).

The overriding structure of the ENISA framework consists of eight major assets: management & organization, network products, organization, processes, services, interconnections, data, and protocols.

Within major asset “Processes” we find “Security Assurance Processes” sub-divided into accreditation, conformity assessment and standardization, as in Figure 2 (A). These latter three assets are divided further into several additional security elements. Also, sub-category Security (B) consists of five elements; and sub-category Security Data (C) is made up of five asset elements.

If we turn to “Network Functions” under major asset “Network Products” we end up at category “Security Functions” which includes Core Network, Network Slice, and NFV Security, each containing additional elements, as can be seen in Figure 2 (C).

Moreover, several of the asset elements contained in Figure 2 (C) appear once more in 5G Core Functions of Figure 3 and attest to the complexity of the cybersecurity architecture inherent in the ENISA 5G framework. These are: SEPP, SEAF, AUSF, ARPF, AMF, UDM, NSSAAF.

What is more, the overall 5G system architecture as put forward in the ENISA report can be considered in terms of the network functions (NFs) outlined in the 3GPP specification ^18^ and clearly depicted in Figure 3. Consider also that a wide range of asset elements in general are positioned throughout the ENISA framework and that these elements are based on the 3GPP specification which in turn is supported by the underlying data contained in the Unified Data Repository ^19^. ^xxx^

**Figure 4.**
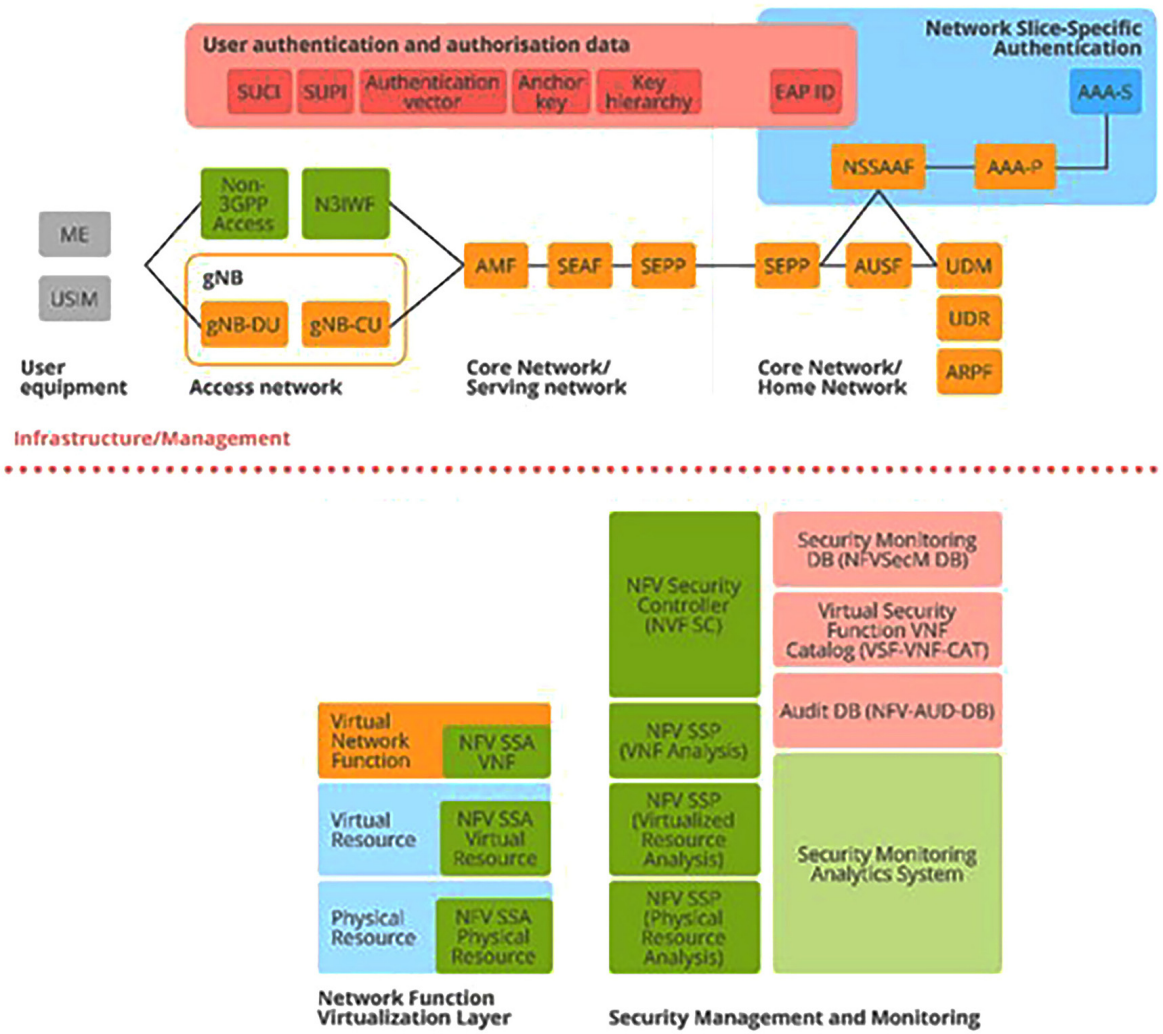
5G Security Architecture. ENISA Threat Landscape Report ( 1, p. 51). Here, the detailed structure of the 5G security architecture is shown and includes various aspects of network security crucial to its smooth functioning: access, signaling, applications, services, visibility, and configurability. These aspects are supported by the assets depicted in Figure 2 and Figure 3 above, where it was shown how these also map to the 5G Security Architecture depicted in this figure. © European Union Agency for Cybersecurity (ENISA), 2020.

Let us now turn to the principal components of ENISA's cybersecurity architecture. These are derived from the 3GPP specification ^4^ and appear prominently in ENISA’s structural model of Section 3.10 5G Security Architecture ( 1, p. 51), as depicted in Figure 4 below. Of primary importance are the authentication and authorization aspects, as depicted in the figure. Subscription authentication is handled by the Subscription Permanent Identifier (SUPI), which calls for agreement between User Equipment (UE) and the network. This also involves the Subscription Concealed Identifier (SUCI).

In addition, the security asset elements that were identified in Figure 2 and Figure 3 as overlapping with each other (SEPP, SEAF, AUSF, ARPF, AMF, UDM, NSSAAF) also appear here in the 5G Security Architecture (top), together with a wide range of Virtual Network Functions (bottom) which map directly to asset elements contained in sub-category NFV Security of Figure 2 (C); and the security elements that appear here in the red, yellow and blue boxes (top) map directly to security asset elements contained in Figures 2 (B) and (C).

This overview of the security related assets and network functions identified by ENISA in relation to security features is just an abstract representation of the architecture of the 5G framework. On a practical level, however, ENISA goes further and provides guidance for competent authorities on how to ensure that appropriate security measures are taken by providers of 5G networks and services. In its 5G supplement to the guideline on security measures ^14^, ENISA clarifies and refines the generic security measures contained in the more general ‘Guideline on Security Measures under the European Electronic Communications Code (EECC) ^13^.’ As stated in its supplementary text: The 5G technology profile gives additional guidance to competent national authorities about how to ensure the security of 5G networks [ 14, p. 5].

## ENISA and European Union / Commission

ENISA’s strategic direction is clearly manifest in a vast array of legislation, directives and reports on cybersecurity put out by the European Union (EU) and/or the European Commission (EC), including a comprehensive cybersecurity strategy. ^xxxi^

The role of ENISA in the EU and EC is unequivocally defined in the Cyber Security Act of 2017 (13), as follows:

The Agency should assist the Commission by means of advice, opinions and analyses on all the Union matters related to policy and law development, update and review in the area of cybersecurity, including critical infrastructure protection and cyber resilience. The Agency should act as a reference point of advice and expertise for Union sector-specific policy and law initiatives where matters related to cybersecurity are involved. ^xxxii^

Moreover, in 2019, the Cybersecurity Act gave ENISA a permanent mandate with additional resources and assigned it new tasks for its operations, including the establishment of a cybersecurity certification framework. ^xxxiii^ In June 2021 the agency established a local office in Brussels which began to give it greater visibility with the EU and EC, seeing that since 2004 ENISA was headquartered in Athens. Its mandate in Brussels is to maintain regular and systematic cooperation with Union institutions and agencies such as the European External Action Service, Europol and the European Defence Agency and other entities involved in cybersecurity. For example, plans include the further development of a Joint Cyber Unit ^xxxiv^ as a virtual platform of cybersecurity tools and a physical platform built around ENISA and Computer Emergency Response Team (CERT-EU) adjacent offices in Brussels. The unit aims to strengthen cooperation among EU institutions, agencies, and various authorities in the Member States.

As for the newly formed European Cybersecurity Competence Centre (ECCC) and Network which aims to strengthen the EU's cybersecurity capacity and competitiveness, ENISA extends activities beyond its corridors to participate on ECCC’s Governing Board via its Executive Director. ^xxxv^

ENISA’s role in cybersecurity is further strengthened by the recent formulation contained in NIS 2 (December 2020), ^xxxvi^ a significant Directive of the EU Parliament that outlines measures for a high level of security of network and information systems across the Union and that advocates for systemic and structural changes to the NIS Directive of 2016. Its preferred policy option includes shared responsibilities and mechanisms aimed at fostering more trust among Member States and authorities and industry, and for information sharing. This option would also “… ensure more involvement of ENISA, within its current mandate, in holding an accurate overview of the cybersecurity state of the Union”. ^xxxvii^

## ENISA in consideration of 3GPP mandatory controls

The mandatory controls specified by 3GPP can be divided into two categories:

i) mandatory controls for implementation are the security measures and protocols that network providers must implement in their 5G networks, while ii) mandatory controls for use are the security measures and practices that users of the network must follow to ensure the security of the network and their own devices.

A careful scan of the segments and sub-segments in Figure 2 and Figure 3 will reveal the distinctions made here between the differing mandatory controls. More specifically, we can detail the distinctions in the following way.

Mandatory controls for implementation: These controls specify the security measures and protocols that network providers must implement in their 5G networks to ensure the security and reliability of the network. These controls are primarily focused on securing the network infrastructure, its elements, and its communication routes.

Examples of mandatory controls for implementation include access control, user authentication, data confidentiality, integrity protection, and network slicing. These controls are implemented by the network providers and service providers.

Mandatory controls for use: These controls specify security measures and practices that users of the network must follow to ensure the security of the network and to protect their own devices and data. These controls are focused on ensuring that devices connected to the network are secure and that users are following proper security protocols and policies.

Examples of mandatory controls for use include password policy, user access control, device authentication, and data encryption. These controls are enforced on the users and devices connected to the network.

ENISA's main role is to support EU member states in enhancing their cybersecurity capabilities and to promote cooperation between EU member states, the private sector, and the research community.

As part of its role, ENISA provides guidance and best practices on cybersecurity, assesses cybersecurity risks, and promotes the development of cybersecurity certification schemes. However, it does not have formal enforcement powers, and its role is mainly advisory and support oriented. Instead, the responsibility of enforcing mandatory use of security controls falls on the member states of the European Union. Each EU member state has its own laws and regulations related to cybersecurity and is responsible for enforcing these laws and ensuring that cybersecurity measures are in place to protect their organizations, citizens, and data.

Although ENISA does not have the power to enforce mandatory use of security controls in the European Union, it can nevertheless advise, as mentioned previously, i.e., ‘give additional guidance to competent national authorities’ [ 14, p. 5].

Moreover, the role of ENISA as an advisory agency, charged with helping or assisting, is forcefully acknowledged in its report on 3GPP and security controls. Consider the following striking statement contained in the report:

The report is also intended to help national competent and regulatory authorities get a better picture of the standardisation environment pertaining to 5G security and to improve understanding of 3GPP security specifications and its main elements and security controls. With this, competent authorities will be in a better position to understand what the key security controls that operators have to implement are and what the role of such controls is for achieving the overall security of 5G networks [ 20, p.7].

## Summary

 The ENISA initiative stands out among other leading-edge 5G projects by its strategic decision to integrate cybersecurity features, along with threats, risks, and vulnerabilities into an all-encompassing architecture from the start of the 5G design and development process.

This strategic approach sets the stage for the implementation of the novel infrastructures, computer models and applications of the future that will need to withstand sophisticated global, hybrid threat onslaughts.

This paper represents the technology component (5G) of a two-part series and should be considered in the context of the second of four principal themes of the H2020 EU-HYBNET project, *i.e.,* ‘Cyber and Future Technologies’. Part 2 will extend the scope of the technology component to include Multi-Access Edge Computing (MEC) capabilities required to deal adequately with hybrid threats in relation to 5G/security and associated issues of concern to EU-HYBNET. Here, ENISA will continue to play a significant role as we take into account its recently released report on security aspects of fog and edge computing in the 5G domain ^21^, as well as the important ENISA study on NFV security in 5G ^17^.

## Ethics and consent 

Ethical approval and consent were not required.

## Data Availability

The paper does not include new data or analysis but takes into account the analysis undertaken by the authors of the ENISA Threat Landscape report ^1^. The data that is examined in the present paper exists in the form of assets described fully by ENISA throughout its report and summarized neatly in a detailed Mind Map, excerpts from which have been included in Figure 2 and Figure 3 above. As well, the assets analyzed by ENISA point to data delineated in the 3GPP specification ^4, 7, 18^ and are based on the Unified Data Repository/UDR Data Storage Architecture, which includes the Unified Data Management (UDM) complex, as described in 19. *Subscription Data* is made available *via* the Unified Data Management (UDM) front-end to a number of Network Functions (NFs) that control the User Equipment (UE) activities within the network: SUCI, SUPI in Figure 4. (top); and AMF, AUSF, NEF, PCF, SMF, etc…as can be viewed in both Figure 3 and Figure 4 (top). Also, the 5G Unified Data Repository (UDR) stores data grouped into distinct collections of subscription-related information. These are made available to various 5G Network Functions (NFs) as can be seen in the excerpt from the ENISA Mind Map above in Figure 3. In summary, ENISA has incorporated the 3GPP data specification – considered by some as a Global Initiative, Mobile Broadband Standard – contained in Release 16 into its 5G design and architecture ^18^ and in its accompanying security features ^4^. In addition, data related to the component ‘Enhancement of Ultra-Reliable and Low Latency Communications (URLLC)’ is referenced in ^7^; and the principal repository of relevance UDR is referenced in ^19^. Finally, a detailed description of how the Unified Data Repository (UDR) serves as a centralized repository for subscription data, subscriber policy data, sessions, contexts, and application states, is contained in Alepo’s 3GPP Release 16 compliant 5G Unified Data Repository (5G UDR – Alepo 5G Core: Unified Data Repository (UDR) | 5G UDR). However, there is no need to consult or access the UDR directly as all data in this study is accessible via the ENISA Threat Landscape Report and the 5G 3GPP specification.

## Footnotes

^i^ Partners (3gpp.org)

^ii^ *Ibid*.

^iii^ GSMA | About the GSMA – Represents the interests of mobile operators worldwide

^iv^ *Ibid*.

^v^ Four 5G RAN vendors are 3GPP/GSMA NESAS compliant | DigiAnalysys

^vi^ eSIM is the generic term used for the embedded form of a SIM (subscriber identity module) card. Although not yet a consumer mass market leader, eSIMs are supported by all major network operators across Europe and by a variety of devices.

^vii^ Release 16 (3gpp.org)

^viii^ https://www.enersec.net/asset-mm/ -- write to 5g.assetmap@enersec.net

^ix^ Vienna & Frankfurt Agreements | EU Project SESEI

^x^ cen-clc_ai_fg_white-paper-response_final-version_june-2020.pdf (cencenelec.eu)

^xi^IMT Vision – Framework and overall objectives of the future development of IMT for 2020 and beyond (itu.int)

^xii^ 5G - Fifth generation of mobile technologies (itu.int)

^xiii^ Coordinated Vulnerability Disclosure: the Guideline | Publication | National Cyber Security Centre (ncsc.nl)

^xiv^ Common Vulnerability Scoring System | NIST

^xv^ CVE - Home (mitre.org)

^xvi^ Software Vulnerability Disclosure in Europe – CEPS

^xvii^ FS.23-v3.0.pdf (gsma.com)

^xviii^ Ibid, pp. 12–15.

^xix^ Hyperconnectivity & IoT | Knowledge for policy (europa.eu)

^xx^ Home - Web of Things (WoT) (w3.org)

^xxi^ Hyperconnectivity & IoT | Knowledge for policy (europa.eu)

^xxii^ GSMA | New GSMA Study: 5G to Account for 15% of Global Mobile Industry by 2025 as 5G Network Launches Accelerate - Newsroom

^xxiii^ Working groups – 5G-ACIA

^xxiv^ 5G Action plan | Shaping Europe’s digital future (europa.eu)

^xxv^ European Electronic Communications Code Updating EU Telecom Rules | Shaping Europe’s digital future (europa.eu)

^xxvi^ 5G-PPP

^xxvii^ Microsoft Word - 5G IA Position Paper - Smart Networks and Services_Horizon Europe_final.docx (5g-ppp.eu)

^xxviii^ The 5G Infrastructure Association (5G IA) publishes the White Paper ‘European Vision for the 6G Network Ecosystem’ ‹ 5G-PPP

^xxix^ Security standards and their role in 5G - Ericsson

^xxx^ What is the 5G Unified Data Repository? UDR | Apis Training

^xxxi^ The Cybersecurity Strategy | Shaping Europe’s digital future (europa.eu)

^xxxii^ Cybersecurity Act, Amendment 16, Proposal for a regulation, Recital 13.

^xxxiii^ The EU Cybersecurity Act | Shaping Europe’s digital future (europa.eu)

^xxxiv^ Joint Cyber Unit | Shaping Europe’s digital future (europa.eu)

^xxxv^ Governing Board (europa.eu)

^xxxvi^ Proposal for directive on measures for high common level of cybersecurity across the Union | Shaping Europe’s digital future (europa.eu)

^xxxvii^ https://eur-lex.europa.eu/legal-content/EN/TXT/?uri=COM:2020:823:FIN, Section 3.
