# Supplementary material for: ENISA: 5G design and architecture of global mobile networks; threats, risks, vulnerabilities; cybersecurity considerations
Source: Open Res Eur. 2023 May 24;2:125. Originally published 2022 Nov 22. [Version 3] doi: 10.12688/openreseurope.15219.3 (PMC10446063; doi:10.12688/openreseurope.15219.3)
Supplement: Peer review history

# Peer review: Reviewer response for version 3

**DOI:** 10.21956/openreseurope.17366.r32030

**Published:** 2023-06-15

**Authors:** Geir M. Køien

**Affiliations:** 1 University of South-Eastern Norway, Horten, Norway

The paper was significantly improved.

Where applicable, are recommendations and next steps explained clearly for others to follow? (Please consider whether others in the research community would be able to implement guidelines or recommendations and/or constructively engage in the debate)

Yes

Does the article adequately reference differing views and opinions?

Partly

Are all factual statements correct, and are statements and arguments made adequately supported by citations?

Partly

Is the rationale for the Open Letter provided in sufficient detail? (Please consider whether existing challenges in the field are outlined clearly and whether the purpose of the letter is explained)

Yes

Is the Open Letter written in accessible language? (Please consider whether all subject-specific terms, concepts and abbreviations are explained)

Yes

Reviewer Expertise:

Security

I confirm that I have read this submission and believe that I have an appropriate level of expertise to confirm that it is of an acceptable scientific standard.

# Peer review: Reviewer response for version 2

**DOI:** 10.21956/openreseurope.17070.r30937

**Published:** 2023-05-11

**Authors:** Franco Callegati

**Affiliations:** 1 Department of Computer Science and Engineering, University of Bologna, Bologna, Italy

The manuscript is well written and provides a review of the 5G Threat landscape report by ENISA. It is a valuable contribution. The analysis of the structure and objectives of the ENISA report is well organized and may be of help to guide potential readers in approaching the document, which is large in size and complex in structure.

 I have some comments on some specific parts:

- Page 4, column 2, paragraph 5. I do not understand what the message of this paragraph is. Apparently there is a mistake in the hyphens, since they are opened once and closed twice, so what part is citation and what is original writing? It looks like the message is that, when there are two many rapporteurs, especially from the same stkeholder, the details of the specification become somehow more fragmented because of too many details. Nonetheless this is not clear. I would suggest to rephrase this paragraph making the message it wants to carry more explicit.
- A similar comment holds for Page 5, column 2, paragraph 3. Again I believe it would be useful to state clearly the scope of this remark. I understand it is a critic about the specification of too discrete standard elements, but the last part of the paragraph is somewhat obscure (achitecture is employed where?).
- Figure 2 shows a low defintion, such that in a printout some lines are not readable. I would suggest to either split it into several figures with larger text or to make some effort to provide it at higher definition. 

Where applicable, are recommendations and next steps explained clearly for others to follow? (Please consider whether others in the research community would be able to implement guidelines or recommendations and/or constructively engage in the debate)

Yes

Does the article adequately reference differing views and opinions?

Yes

Are all factual statements correct, and are statements and arguments made adequately supported by citations?

Yes

Is the rationale for the Open Letter provided in sufficient detail? (Please consider whether existing challenges in the field are outlined clearly and whether the purpose of the letter is explained)

Yes

Is the Open Letter written in accessible language? (Please consider whether all subject-specific terms, concepts and abbreviations are explained)

Yes

Reviewer Expertise:

5G, SDN, NFV

I confirm that I have read this submission and believe that I have an appropriate level of expertise to confirm that it is of an acceptable scientific standard.

# Response

**Published:** 2023-05-16

**Authors:** R. Andrew Paskauskas

**Affiliations:** Lithuanian Cybercrime Center of Excellence for Research,Training and Education, Lithuania

Thank you, Professor Callegati, for taking the time to provide a review of my ENISA 5G paper. Your efforts are much appreciated. I have taken into account your comments and revised accordingly, eliminating the ambiguous sentences that you have referred to, as well as submitting a higher resolution Figure 2. My adjustments will appear in Version 3.

# Peer review: Reviewer response for version 1

**DOI:** 10.21956/openreseurope.16460.r30935

**Published:** 2023-03-17

**Authors:** Geir M. Køien, Shahzana Liaqat

**Affiliations:** 1 University of South-Eastern Norway, Horten, Norway

The topic is relevant and there is no doubt about the importance of the ENISA reports. However, the article lacks clarity and focus in some areas. In particular, the article title gives the impression that this is a survey of ENISA efforts towards 5G security. While it is that, the article also provides a lot of coverage on the history of the standardization and the various standardization bodies that are involved with or are affect by the mobile security standards. Most of this text is somewhat superfluous.

 It must be noted that the article is misleading in some areas. For instance, the *Group Speciale Mobile*, did not evolve into GSMA as alluded to in page 3 in the PDF (and in Table III). That group was a CEPT group. However, the historical role of CEPT is scarcely important for the current 5G standards.

*Our advice is that all material that isn’t directly relevant to 5G and/or ENISA is reconsidered. It distracts from the main message, and some of it seems irrelevant.*

 We also note that the ITU-R is the body that defined the high-level requirements for 5G. Thus, it was not really the 3GPP that defined the “5G Use Cases” (page 4). These use-cases are directly derived from the triangle vision (see ITU-R M.2083-0 “..IMT for 2020 and beyond” 09/2015). On page 6, the texts seem to indicate that ITU-R “relied heavily on the 3GPP specifications.”. However, it is ITU-R that defined the 5G vision. The 3GPP works out how to meet the goals of the IMT visions. And, the ITU has a UN mandate to define these visions and high-level requirements.

 Now, concerning ENISA’s role in 5G security, it should be mentioned that the 5G Threat Landscape report is part of a series of Threat Landscape reports, which features annual and topical reports. Additionally, ENISA have also published other 5G relevant reports, including:

- “5G Cybersecurity Standards”, https://www.enisa.europa.eu/publications/5g-cybersecurity-standards
- “5G Supplement - to the Guideline on Security Measures under the EECC”, https://www.enisa.europa.eu/publications/5g-supplement-security-measures-under-eecc
- Embedded Sim Ecosystem, Security Risks and Measures[1], https://www.enisa.europa.eu/publications/embedded-sim-ecosystem-security-risks-and-measures
- “NFV Security in 5G - Challenges and Best Practices”, https://www.enisa.europa.eu/publications/nfv-security-in-5g-challenges-and-best-practices
- “Security in 5G Specifications - Controls in 3GPP”, https://www.enisa.europa.eu/publications/security-in-5g-specifications
- “Fog and Edge Computing in 5G”, https://www.enisa.europa.eu/publications/fog-and-edge-computing-in-5g

These ENISA works should surely be mentioned given the title of the article.

 We find the section discussing “Cybersecurity considerations” somewhat contrived with respect to the roles of the various bodies and groups. The 3GPP SA3 is indeed instrumental in defining the cybersecurity for the mobile systems. However, the 3GPP produces technical specifications and technical reports. Cybersecurity is sometimes sensitive, and there are different legal and regulatory regimes that affect which security controls are actually put into use. The 3GPP has many specifications with associated requirements. Then, there are many requirements that are “mandatory for implementation”, but still not “mandatory for use”.

 Here, ENISA has a very important position as an EU agency. ENISA advise can effectively turn security controls into “mandatory” for use (within the EU jurisdictional reach).

 The paragraphs discussing Huawei (page 10) seems superfluous.

 Figures 2 and 3 are not legible.

 We recommend that the article is revised and corrected as need be.

Where applicable, are recommendations and next steps explained clearly for others to follow? (Please consider whether others in the research community would be able to implement guidelines or recommendations and/or constructively engage in the debate)

Yes

Does the article adequately reference differing views and opinions?

Partly

Are all factual statements correct, and are statements and arguments made adequately supported by citations?

Partly

Is the rationale for the Open Letter provided in sufficient detail? (Please consider whether existing challenges in the field are outlined clearly and whether the purpose of the letter is explained)

Yes

Is the Open Letter written in accessible language? (Please consider whether all subject-specific terms, concepts and abbreviations are explained)

Yes

Reviewer Expertise:

Cybersecurity, mobile systems

We confirm that we have read this submission and believe that we have an appropriate level of expertise to confirm that it is of an acceptable scientific standard, however we have significant reservations, as outlined above.

# Response

**Published:** 2023-03-21

**Authors:** R. Andrew Paskauskas

**Affiliations:** Lithuanian Cybercrime Center of Excellence for Research,Training and Education, Lithuania

Thank you for your comments, insights and suggestions for revision. I have seriously taken these into account and have revised accordingly where appropriate. Here is my response to your review. The opening paragraph concludes with the statement: “…the article … provides a lot of coverage on the history of the standardization and the various standardization bodies that are involved with or are affect[ed] by the mobile security standards. Most of this text is somewhat superfluous.” I point out that Reviewer No. 1. has a different view of this issue, which coincides with mine. She writes: “…many standardization and related-to-standadization organizations mainly focus on specific aspects, which although relevant to the European single market and to securing the business interests of stakeholders, lack a comprehensive and holistic view …”  Thus, I am tempted to retain this part of my text because of ENISA’s painstaking efforts to stand out among other leading-edge players in the 5G and standards arena and achieve its strategic aims of integrating cybersecurity considerations with threats, risks, and vulnerabilities into an architecture of 5G right from the start of the design and development process. ENISA's approach differs significantly from the status quo, I think. Further on the following suggestion is made: “ *Our advice is that all material that isn’t directly relevant to 5G and/or ENISA is reconsidered. It distracts from the main message, and some of it seems irrelevant.*” I am willing to eliminate much of the text that is irrelevant as identified subsequently in the review. i) The review points out: “For instance, the *Group Speciale Mobile*, did not evolve into GSMA as alluded to in page 3 in the PDF (and in Table III). That group was a CEPT group. However, the historical role of CEPT is scarcely important for the current 5G standards.” I would like to point out that the segment on ‘Brief History of GSM & the GSMA’ [ https://www.gsma.com/aboutus/history ] does lead the reader to conclude that *Groupe spéciale mobile* (GSM) did indeed evolve into the GSMA. The history of the GSM and GSMA can be traced through the years 1982, when GSM was associated with CEPT, up to 1987, when the MoU was signed forming GSMA, through 1989, 1993, and up to 1995 when we have explicit mention of the MoU and GSMA alongside GSM. In fact, the ENISA report on Fog and Edge computing [ https://www.enisa.europa.eu/publications/fog-and-edge-computing-in-5g ] refers to ‘The *Groupe Spécial Mobile* Association (GSMA) …’ p. 19; although elsewhere on the Internet, but not on the GSMA web site, we can uncover the expanded association name ‘Global System for Mobile Communications Association (GSMA).’ I will employ this latter English version in the revised version of the paper. ii) The comments on the role of ITU-R in relation to 3GPP (page 6 of the paper) are valid and I have modified my text, accordingly.  As for the observation “…it was not really the 3GPP that defined the “5G Use Cases” (page 4),” I point out that it may indeed be that ITU-R did originally ‘define’ these use cases, as suggested, but this segment of the paper merely indicates that ENISA is putting forward these 5G Use Cases as being based on the 3GPP Release 16 specification. I have made a small change in the text to emphasize this latter point. The suggestion that the ENISA 5G Threat Landscape report is part of a series of Threat Landscape reports has been acknowledged in the opening paragraph of the paper. The other 5G relevant reports listed have been identified and elaborated on throughout the paper at appropriate points in the argument. Next point: “We find the section discussing 'Cybersecurity considerations' somewhat contrived with respect to the roles of the various bodies and groups.” I agree to cut the segment on ‘The United States’, but keep the segment on Europe, where I am inserting some text related to ENISA. The comments on 3GPP security controls are relevant, especially the distinction made between “…mandatory for implementation versus mandatory for use” and the role that ENISA could take on in this context. I am including a lengthy insertion on this aspect, which appears towards the end just before the Summary. The suggestion that “The paragraphs discussing Huawei (page 10) seems superfluous” is valid and I am eliminating these. I have managed to fix up Figures 2 and 3 and am submitting two new files in 300 dpi and TIFF format, but remaining work still is in the hands of ORE’s production team.     

# Peer review: Reviewer response for version 1

**DOI:** 10.21956/openreseurope.16460.r30452

**Published:** 2023-01-09

**Authors:** Marina Settembre

**Affiliations:** 1 Fondazione Ugo Bordoni, Rome, Italy

The paper outlines the leading-edge role played by the European Union Agency for Cybersecurity (ENISA) in the 5G arena, by highlighting ENISA's painstaking activity in formulating an integrated framework of 5G network architecture. The ENISA's 5G framework, based on 3GPP specifications, integrates vulnerabilities, threats, risks, and cybersecurity considerations in a particularly complex and fragmented scenario. In fact, many standardization and related-to-standadization organizations mainly focus on specific aspects, which although relevant to the European single market and to securing the business interests of stakeholders, lack a comprehensive and holistic view, except for a few cases. In particular, the author guides the reader in analyzing the ENISA’s remarkable and information-dense report (i.e. Enisa Threat Landscape - December 2020), integrating his helpful comments to better grasp some key aspects.

 The paper is well structured and sufficiently clear in the exposition, particularly considering that the topic is very vast and multi-faceted. It might also be interesting to highlight the relevance of a controlled and shared taxonomy, to which ENISA with its work contributes significantly, to develop ontology-based approaches for a shared knowledge base and reasoning. Ontology is not the final step, but the basis for useful applications and tools in complex, heterogeneous and dynamic scenarios that will increasingly need some automation such as 5G cybersecurity and management domains. 

 The reference literature, as also pointed out by the author himself, is truly vast. The paper directs the reader to recent studies containing themselves many pages and references. However, considering the topics addressed in the paper, I would suggest that the author also consider including a reference to another report by Enisa: 5G Cybersecurity Standards, March 2022 (https://www.enisa.europa.eu/publications/5g-cybersecurity-standards). This report focuses on standardization from a technical and organizational perspective and provides an interesting assessment of coverage and evaluation of gaps in 5G standardization and recommendations. 

 In the concluding summary the author announces a future in-depth study on Multi-Access Edge Computing (MEC), not covered in the present paper. In that context, I guess the author will refer to another relevant Enisa report - NFV Security in 5G: Challenges and Best practices, February 2022 - not included in this paper.

Where applicable, are recommendations and next steps explained clearly for others to follow? (Please consider whether others in the research community would be able to implement guidelines or recommendations and/or constructively engage in the debate)

Yes

Does the article adequately reference differing views and opinions?

Yes

Are all factual statements correct, and are statements and arguments made adequately supported by citations?

Yes

Is the rationale for the Open Letter provided in sufficient detail? (Please consider whether existing challenges in the field are outlined clearly and whether the purpose of the letter is explained)

Yes

Is the Open Letter written in accessible language? (Please consider whether all subject-specific terms, concepts and abbreviations are explained)

Yes

Reviewer Expertise:

Cybersecurity, 5G networks

I confirm that I have read this submission and believe that I have an appropriate level of expertise to confirm that it is of an acceptable scientific standard.

# Response

**Published:** 2023-03-21

**Authors:** R. Andrew Paskauskas

**Affiliations:** Lithuanian Cybercrime Center of Excellence for Research,Training and Education, Lithuania

Thank you for your review. I have given your suggestions serious consideration, in particular ‘the relevance of a controlled and shared taxonomy, to which ENISA with its work contributes significantly.’  However, it may take some time and effort ‘to develop ontology-based approaches for a shared knowledge base and reasoning,’ but I will definitely give this careful thought. In the 2 ^nd^ version of the present paper, I have also included mention of the ENISA report on 5G Cybersecurity Standards that you refer to, as well as the other ENISA 5G reports of which there are five more, including your suggested report on NFV Security in 5G: Challenges and Best practices, which will also be given prominence in Part II on MEC.
